# Complete Genome Sequence of a Listeria monocytogenes Strain Isolated from Sprouts and Carrying an Antimicrobial Resistance Gene

**DOI:** 10.1128/mra.00200-22

**Published:** 2022-06-06

**Authors:** Hongsheng Huang, Amit Mathews, Marc-Olivier Duceppe, Mingsong Kang, Lin Ru Wang, Sohail Naushad

**Affiliations:** a Ottawa Laboratory—Fallowfield, Canadian Food Inspection Agency, Ottawa, Ontario, Canada; b Greater Toronto Area Laboratory, Canadian Food Inspection Agency, Toronto, Ontario, Canada; University of Arizona

## Abstract

Listeria monocytogenes, a Gram-positive, rod-shaped, non-spore-forming bacterium, is an important foodborne bacterial pathogen for humans worldwide, with a high mortality rate. Here, we report the complete genome sequence of a Listeria monocytogenes strain with an antimicrobial resistance (AMR) gene, isolated from sprouts in Canada.

## ANNOUNCEMENT

The Gram-positive organism Listeria monocytogenes, found in various environments, animals, and food, including bean sprouts, is one of the most important foodborne pathogens for humans worldwide, with a high mortality rate (1). In light of the rarity of publications of complete genome sequences of L. monocytogenes isolates from sprouts, we report the complete genome sequence of a L. monocytogenes GTA-L201 strain (also referred to as CFIAFB20140043) isolated from sprouts grown and collected in refrigerated closed bags from a retail in Canada in April 2014. Fifty grams of sprouts were stomached for 1 minute (setting 7) using Bagmixer 400 (Interscience, USA) in 450 ml of *Listeria* enrichment broth - UVM (University of Vermont Medium) formulation for primary enrichment (30°C, 24 hours), and subsequent incubation in MOPS (morpholinepropanesulfonic acid)-Buffered *Listeria* Enrichment Broth for secondary enrichment (35°C, 24 hours). The MOPS-BLEB was streaked onto RAPID *L.mono* and Oxford agar plates with incubation at 35°C for 24 hours. The *Listeria*-suspect colonies were confirmed by hemolysis on blood agar plates (35°C, 24 hours) and rapid Vitek (bioMérieux, Canada) (2). A single colony was isolated for further genomic DNA extraction.

Genomic DNA (gDNA) was extracted from an overnight culture of GTA-L201 in brain heart infusion medium using a Maxwell 16 cell DNA purification kit (Promega, USA) for Illumina sequencing and a NanoBind CBB Big DNA kit (Circulomics, USA) for Nanopore sequencing, with quantification using a Qubit fluorometer (Thermo Fisher Scientific, USA). Illumina MiSeq sequencing was conducted by library preparation (Nextera XT kit; Illumina, USA) followed by sequencing for 300 bp cycles. Nanopore sequencing was performed using a one-dimensional native barcoding gDNA protocol (EXP-NBD104 and SQK-LSK109; Oxford Nanopore Technologies, UK) without shearing followed by sequencing using a FLO-MIN106 (R9.4.1) flow cell on a MinION Mk1C device. Nanopore sequencing base calling was performed using Super Accuracy mode in Guppy v5.0.11, trimming using Porechop v0.2.3 (3), and filtration using Filtlong v0.2.1 (4). Assembly of the Nanopore long reads was performed using Flye v2.7 (5), corrected using Medaka v1.4.4, and polished with Illumina MiSeq reads using a combination of NextPolish v1.4.0, ntEdit v1.3.5, and Polypolish v0.5.0 after trimming/filtering with fastp v0.23.2. The circularity and genome rotation using *dnaA* as the starting point were determined using the fixstart plugin from Circlator v1.5.5 (6). The sequencing coverage depth was determined using Minimap2 v2.17 (7) and SAMtools v1.13 (8) for long reads and BWA v0.7.17 and SAMtools for short reads. Gene prediction and annotation were performed using the NCBI Prokaryotic Genome Annotation Pipeline (PGAP) v6.0 (9).

Antimicrobial resistance (AMR) genes were identified using ResFinder v4.1.5 (10) and RGI v5.2.0 (11). The plasmids were identified by mlplasmids v1.0.0 (12). Prophage sequences were analyzed using PHASTER (13), accessed online on 31 January 2022. Default parameters of the above bioinformatics pipelines were used except where otherwise noted (https://github.com/OLF-Bioinformatics/nanopore).

The MiSeq had 752,918 paired-end raw reads and 100× coverage, and Nanopore sequencing had 79,499 raw reads, 329× coverage and N50 ≥ 35,544 bp with the longest read of 203,526 bp. The GTA-L201 genome contains a single chromosome (2,910,079 bp), harboring a *fosX* AMR gene, without identification of plasmid and intact prophages (PHASTER score, >90). The annotated genome contains 2,778 proteins and 67 tRNAs with 38.01 GC%, which are similar to the 2,889 coding sequences (CDS) and 37.88 GC% for L. monocytogenes on average in GenBank (accessed on 31 January 2022).

### Data availability.

The whole-genome sequence of GTA-L201 was deposited in GenBank under accession number CP092061. MinION base-called and MiSeq base-called fastq files are available in the NCBI Sequence Read Archive (SRA) under accession numbers SRR17965226 and SRR17965218, respectively.
